# From Pediatric to Adult Brain Cancer: Exploring Histone H3 Mutations in Australian Brain Cancer Patients

**DOI:** 10.3390/biomedicines11112907

**Published:** 2023-10-27

**Authors:** Benedicte Grebstad Tune, Heena Sareen, Branka Powter, Smadar Kahana-Edwin, Adam Cooper, Eng-Siew Koh, Cheok S. Lee, Joseph W. Po, Geoff McCowage, Mark Dexter, Lucy Cain, Geraldine O’Neill, Victoria Prior, Jonathan Karpelowsky, Maria Tsoli, Lars O. Baumbusch, David Ziegler, Tara L. Roberts, Paul DeSouza, Therese M. Becker, Yafeng Ma

**Affiliations:** 1 Department of Pediatric Research, Division of Paediatric and Adolescent Medicine, Oslo University Hospital Rikshospitalet, 0372 Oslo, Norway; b.g.tune@studmed.uio.no (B.G.T.); lars.o.baumbusch@rr-research.no (L.O.B.); 2 Institute of Clinical Medicine, University of Oslo, 0318 Oslo, Norway; 3 Centre for Circulating Tumour Cell Diagnostics and Research, Ingham Institute for Applied Medical Research, 1 Campbell St, Liverpool, NSW 2170, Australia; h.sareen@student.unsw.edu.au (H.S.); branka.powter@health.nsw.gov.au (B.P.); adam.cooper@health.nsw.gov.au (A.C.); joseph.po@hotmail.com (J.W.P.); tara.roberts@westernsydney.edu.au (T.L.R.); paul.desouza@sydney.edu.au (P.D.); yafeng.ma@unsw.edu.au (Y.M.); 4 South Western Sydney Clinical School, University of New South Wales, Goulburn St, Liverpool, NSW 2170, Australia; engsiew.koh@health.nsw.gov.au; 5 Children’s Cancer Research Unit, Kids Research, The Children’s Hospital at Westmead, Westmead, NSW 2145, Australia; smadar.kahanaedwin@health.nsw.gov.au (S.K.-E.); geraldine.oneill@health.nsw.gov.au (G.O.); victoria.prior@hotmail.com (V.P.); jonathan.karpelowsky@health.nsw.gov.au (J.K.); 6 School of Medicine, Western Sydney University, Campbelltown, NSW 2560, Australia; soon.lee@westernsydney.edu.au; 7 Department of Radiation Oncology, Liverpool Hospital, Liverpool, NSW 2170, Australia; 8 Cancer Centre for Children, The Children Hospital at Westmead, Westmead, NSW 2145, Australia; geoff.mccowage@health.nsw.gov.au (G.M.); cainl4@chop.edu (L.C.); 9 Neurosurgery, The Children Hospital at Westmead, Westmead, NSW 2145, Australia; mark.dexter@health.nsw.gov.au; 10 The University of Sydney Children’s Hospital Westmead Clinical School, Faculty of Medicine & Health, The University of Sydney, Westmead, NSW 2145, Australia; 11 Paediatric Oncology and Thoracic Surgery, The Children’s Hospital at Westmead, Westmead, NSW 2145, Australia; 12 Division of Child and Adolescent Health, The University of Sydney, Camperdown, NSW 2050, Australia; 13 Children’s Cancer Institute, Randwick, NSW 2031, Australia; maria.tsoli@unsw.edu.au (M.T.); d.ziegler@unsw.edu.au (D.Z.); 14 Faculty of Health, Welfare and Organization, Østfold University College, 1757 Halden, Norway; 15 Kids Cancer Centre, Sydney Children’s Hospital, Randwick, NSW 2052, Australia; 16 School of Clinical Medicine, UNSW Medicine & Health, UNSW Sydney, Kensington, NSW 2052, Australia

**Keywords:** childhood brain cancer, adult glioma, glioblastoma, circulating free DNA (cfDNA), circulating tumor DNA (ctDNA), histone mutations, droplet digital PCR (ddPCR)

## Abstract

Genetic histone variants have been implicated in cancer development and progression. Mutations affecting the histone 3 (H3) family, H3.1 (encoded by *HIST1H3B* and *HIST1H3C*) and H3.3 (encoded by *H3F3A*), are mainly associated with pediatric brain cancers. While considered poor prognostic brain cancer biomarkers in children, more recent studies have reported H3 alterations in adult brain cancer as well. Here, we established reliable droplet digital PCR based assays to detect three histone mutations (H3.3-K27M, H3.3-G34R, and H3.1-K27M) primarily linked to childhood brain cancer. We demonstrate the utility of our assays for sensitively detecting these mutations in cell-free DNA released from cultured diffuse intrinsic pontine glioma (DIPG) cells and in the cerebral spinal fluid of a pediatric patient with DIPG. We further screened tumor tissue DNA from 89 adult patients with glioma and 1 with diffuse hemispheric glioma from Southwestern Sydney, Australia, an ethnically diverse region, for these three mutations. No histone mutations were detected in adult glioma tissue, while H3.3-G34R presence was confirmed in the diffuse hemispheric glioma patient.

## 1. Introduction

Histones act as spools around which eukaryotic DNA is organized and are critical for regulating access to DNA for replication, transcription, and repair. As such, histones are associated with epigenetic regulation of the genome, and it is not surprising that mutations/alterations are implicated in cancer development and progression. Indeed, histone mutations are common in tumors affecting children and adolescents and are associated with poorer overall survival (OS) [1,2,3]. Histone 3 (H3) is the most frequently mutated histone in cancer, initially identified in pediatric high-grade glioma in 2012 [4,5]. Subsequently, these mutations have been identified in a spectrum of other cancer types, including chondroblastoma, head and neck squamous cell carcinoma, and acute myeloid leukemia [6,7,8]. Notably, most of these point mutations are predominantly found in histone variant H3.3 (encoded by *H3F3A/B*), while mutations in the canonical histone H3.1 (primarily on *HIST1H3B*) are less common in kids and are rare in adults. Specific histone mutations are found at a higher frequency in certain tumors [6,9] predominantly affecting children and young adults, such as the K27M alteration in both H3.1 and H3.3 and the G34R/V mutations in H3.3. These alterations have, since their discovery, given rise to classifying two types of distinct tumor entities: “diffuse midline glioma (DMG), H3-K27-altered” and “diffuse hemispheric glioma (DHG), H3-G34-mutant” [10]. While traditionally linked to pediatric brain cancer, recently, histone alterations in adult brain cancer have been increasingly recognized [11,12,13]. 

Being the most common histone mutation, H3.3-K27M is detected in approximately 80% of pediatric diffuse midline glioma (DMG), while the less frequent H3.1-K27M mutation accounts for most of the remaining DMG cases. The H3 mutations display distinct clinical characteristics. For instance, H3.1-K27M tumors are primarily enriched in the pons, typically manifesting at an earlier age (5 years), and correlate with an increased overall survival (15 months) compared to the other H3 tumors. In contrast, H3.3-K27 mutations are predominantly restricted to midline regions with an age of onset typically between 7 and 10 years, and shorter overall survival (10 months) [14]. Studies undertaken thus far indicate that the clinical characteristics caused by histone mutations differ for adult brain cancer [15], such as the primary tumor site, which is most commonly located in the brainstem of pediatric patients while being most commonly found in the thalamus and spinal cord in adults [16]. Adult brain cancer histone mutations are also generally identified in younger patients. Lowe et al. found that the median age of diagnosis was 32 years (age range 18–82) [9]. Similarly, Bonner et al. found adult central nervous system (CNS) cancer histone alterations predominantly in adolescent and young adult (AYA) patients, including those with glioblastoma (GBM) [17]. AYA patients are generally defined as those between 15 and 39 years of age [18].

DHG, characterized by the H3.3-G34R/V mutation in the cerebral hemisphere, is mostly found in slightly older pediatric patients (around 15 years) but is rare among adults. Consequently, data on these tumors in adults are scarce, yet reports suggest that both pediatric and adult patients with this alteration generally have better OS compared to H3-K27M mutation carriers, possibly due to the more amenable locations of the associated tumors for surgical resections [1,19]. Overall, uncertainties remain regarding histone mutations in adult brain cancer.

In brain cancer, the accurate identification of biomarkers poses challenges, primarily attributed to the intricate location of brain tumors, which often makes it difficult or even impossible to obtain tissue biopsies for analysis. Considering these difficulties, researchers have turned their attention to a promising non- or less-invasive alternative known as liquid biopsy, which allows access to tumor-derived information and material from body fluids. Tumors release both whole cells and parts thereof, such as circulating tumor DNA (ctDNA), into body fluids, such as blood and cerebrospinal fluid (CSF), which can be used to monitor tumor evolution and treatment response without exposing the patient to the unnecessary risks that standard tissue biopsies can entail [20].

We recently developed three highly sensitive and specific droplet digital PCR (ddPCR) assays, shown to detect as low as one mutant DNA template in a background of ≥1000 wild-type molecules (assay sensitivity of mutation detection: 0.1%), designed to detect the most common histone H3 mutations, H3.1-K27M, H3.3-K27M, and H3.3-G34R, in tumor tissue or liquid biopsies. Since distinct adult glioma mutations may affect patient prognosis and because mutation status may vary depending on the patient’s ethnicity, we decided to screen a cohort of 89 glioma patients plus a young H3-G34-mutant DHG patient from our ethnically diverse Southwestern Sydney Local Health District in Australia for these histone mutations previously linked to childhood brain cancer.

## 2. Materials and Methods

### 2.1. Patient Samples

Surgical tissue samples from 90 adult glioma patients (89 most diagnosed with GBM prior 2021 WHO classification [10], 1 diagnosed as H3-G34-mutant DHG), treated at Liverpool Hospital in the period from 2013 to 2021 were acquired from the Centre of Oncology Education and Research Translation (CONCERT) biobank. Written consent was obtained from all patients. The study was conducted under research protocols approved by the Southwestern Sydney LHD Human Ethics Committee (HREC13/LPOOL/158 and HREC12/LPOOL/252). DNA was isolated from fresh frozen tissue using QIAamp DNA mini kit (Qiagen, Clayton, Australia) following manufacturer’s instructions and eluted in 200 µL of TE buffer. A Qubit fluorometer was used to determine the purity and concentration of DNA. Liquid biopsies for two patients diagnosed with DIPG (aged 2.5 and 3.3 years) and collected at the Children’s Hospital at Westmead under research protocols approved by the Sydney Children’s Hospitals Network Human Research Ethics Committee (HREC/17/SCHN/302) were also included, with patients’ parents consent, to be part of the study. Histone mutation profiling had been performed for DIPG Patient 1 as a part of the Biomede study (ClinicalTrials.gov Identifier: NCT02233049), which identified the H3.1-K27M variant. Three mL blood samples were collected in 10 mL Cell-Free DNA Streck tubes (Cell-Free DNA BCT^®^, STRECK, La Vista, NE, USA, catalog No. 218997) for both patients at diagnosis and post-treatment (radiotherapy alone or in combination with erlotinib). Blood samples were double spun: 1600 g for 10 min followed by plasma supernatant aspiration into new tubes without disturbing the buffy coat layer, then 15,500 g for 10 min followed by aspirating the top phase into new tubes without disturbing the pellet and storing at −80 until DNA isolation. Two 100–200 µL CSF samples without additives were also collected for DIPG patient 1, two days apart during treatment. The first CSF sample was taken during stereotactic insertion of a ventriculoperitoneal shunt (and processed in the lab within 15 min), the second, during the ventriculoperitoneal shunt revision and catheter change, was processed within 35 min. CSF samples were also double centrifuged, first at 300× *g* for 10 min, then at 15,500× *g* for 10 min, followed by top phase aspiration into new tubes and storage at −80 °C until DNA isolation. Cell-free DNA (cfDNA) was extracted from 1 mL plasma or 100–200 µL CSF using the QIAamp nucleic acid extraction kit (Qiagen, Clayton, Australia) and eluted into 100 µL elution buffer. A 2 µL sample of cfDNA was used per reaction as a ddPCR template. 

### 2.2. DIPG Cell Culture

Primary brain tumor cultures were grown in stem cell media containing DMEM/F12 and Neurobasal medium (Invitrogen, Waltham, MA, USA) (50:50) supplemented with glutamax, pyruvate, non-essential amino acids, HEPES buffer, and antibiotic/antimycotic (Invitrogen). During culture passage, stem cell media were freshly supplemented each time with heparin (Stem Cell Technologies, Tullamarine, Australia), human EGF, and human basic FGF (Stem Cell Technologies). DIPG cultures were also supplemented with PDGF-AA and PDGF-BB (Stem Cell Technologies). In some cultures, 5% of fetal calf serum was added. Cultures were maintained at 37 °C in a humidified atmosphere with 5% CO_2_. HSJD-DIPG007 and HSJD-GBM2 were kindly provided by Dr. A. Montero-Carcaboso (St John of Hope Hospital, Barcelona, Spain), whereas SU-DIPG cultures and VUMC-DIPG10 were generously given by Prof M. Monje (Stanford University, Stanford, CA, USA) and Prof E. Hulleman (Amsterdam University Medical Center, Amsterdam, The Netherlands), respectively. The remaining cultures were established as part of the Zero Childhood Cancer/PRISM personalized medicine clinical trial using the described culture conditions.

### 2.3. Cell-Free Conditioned Media

Since cfDNA is also released by cultured cells, conditioned media can be used as a surrogate for liquid biopsy for the validation of ddPCR assays. Cell-free media, conditioned by use for culture of DIPG cell lines with known histone mutation status, was harvested from primary DIPG cells that were undergoing expansion and were then processed similar to the CSF processing: 300× *g* for 10 min to remove any residual cells. The supernatant was spun again at 15,500× *g* for 10 min to remove cell fragments and debris. All centrifugation steps were performed at room temperature. DNA was extracted from 3 mL media using the QIAmp Circulating Nucleic acid extraction kit (Qiagen, Clayton, Australia) according to the manufacturer’s instructions and eluted in 50 µL elution buffer. Samples were diluted between 1:50 and 1:500 for sensitivity testing and 2 µL was used as a template for the ddPCR assays. 

### 2.4. Primers, Probes and Synthetic gBlock DNA 

Forward and reverse primer sequences, probes, and synthetic DNA fragments were designed for assay optimization and are listed in Table 1 (purchased from Integrated DNA Technologies, Singapore).

### 2.5. H3.3-K27M, H3.3-G34R and H3.1-K27M ddPCR Assays

Synthetic double-stranded DNA fragments (gBlocks of 147–172 base pair length (see Table 1) from Integrated DNA Technologies, Singapore) carrying the studied mutations were used for ddPCR assay optimization. As templates, 2 × 10^−4^ pg of gBLock DNA was mixed with 2 µL of 1 ng/µL genomic DNA (gDNA), extracted from healthy donor human peripheral mononuclear cells, mimicked heterozygous DNA. Each ddPCR reaction contained 250 nM probes and 500 nM primers and 2X ddPCR Supermix for probes, no dUTP (Bio-Rad, South Granville, Australia) and ddPCR reactions were performed using the QX200 ddPCR set up (BioRad). PCR conditions were as follows: 10 min 95 °C, plus 40 cycles of 30 s 94 °C, 1 min 57 °C; followed by 10 min 98 °C; and data were analyzed using the QuantaSoft software, version 1.7.4.0917, to detect mutations (FAM) and wild-type (HEX) droplets. Thresholds to allow for specific mutation and wild-type detection were set manually. 

## 3. Results

**Figure 1:**
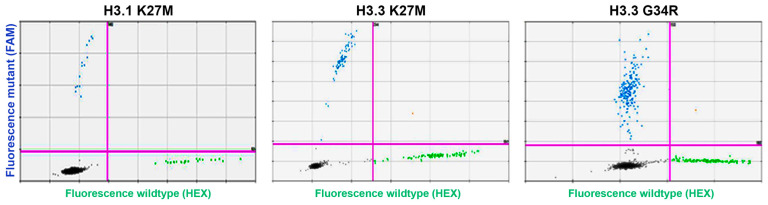
**ddPCR histone mutation assay**. Mutant synthetic gBlock DNA mixed with genomic DNA from healthy donor PBMCs was used as a template for ddPCR. Clear separation of the various indicated mutant and wild-type alleles is demonstrated. Green dots define droplets with wild-type template detected, blue dots define droplets with the indicated mutant detected.

We developed ddPCR assays that achieved successful distinction of mutant and wild-type alleles for H3.3-K27M, H3.3-G34R, and H3.1-K27M, initially using synthetic DNA fragments containing the relevant mutations and a mix of these mutant template DNAs within gDNA from healthy donor PBMCs (Figure 1). The assays were further validated and demonstrated highly specific mutation detection in DNA isolated from conditioned media (as cfDNA equivalent) of established DIPG/brain cancer cell lines with known mutation status (Table 2). 

**Figure 2:**
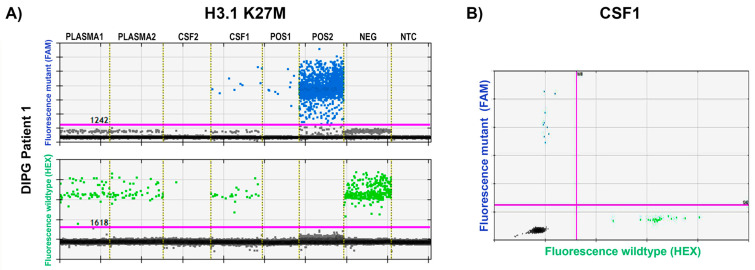
**H3.1-K27M detection in DIPG patient 1**. (**A**) 1D graphical data of a H3.1-K27M assay is shown for ctDNA samples from DIPG patient 1, extracted from plasma and cerebral spinal fluid (CSF) alongside synthetic gBlock template H3.1-K27M DNA (positive control) concentrated at 2 × 10^−4^ pg (POS2) or 1000-fold diluted (POS1), genomic DNA from healthy donor PBMCs (negative control) was used independently to demonstrate wild-type (WT) allele detection. Visual separation of samples by dotted line. Plasma 1—at diagnosis; Plasma 2—post erlotinib and radiotherapy treatment; CSF1—during erlotinib and radiotherapy treatment; CSF2—during erlotinib and radiotherapy treatment, two days after CSF1; POS1—positive control 1, 1:1000 diluted; POS2—positive control 2 1:1; NEG—negative control (healthy donor PBMCs); NTC—non-template control. (**B**) 2D graph of H3.1-K27M detection in the CSF1 sample taken during erlotinib and radiotherapy treatment. Green dots define droplets with wild-type template detected, blue dots define droplets with the indicated mutant (H3.1-K27M) detected.

Since histone mutations are frequently present in pediatric diffuse intrinsic pontine glioma (DIPG, or diffuse midline glioma as reclassified by the World Health Organization [10]), we tested available liquid biopsies from two DIPG patients. For DIPG patient 1 (Biomede study had detected the H3.1-K27M mutation), we tested cfDNA extracted from plasma collected at diagnosis and post treatment with erlotinib and radiotherapy, as well as two CSF samples collected during the same treatment regime two days apart. Wild-type was detected in all plasma samples, suggesting successful quality cfDNA extraction; however, mutant DNA was not detected in either of the plasma cfDNAs. Further, while the H3.1-K27M mutation was readily detectable and validated in the first CSF sample, this mutation was not detected in the CSF sample taken two days later. Of note, the assay only detected minimal amounts of wild-type DNA in this specific sample, suggesting that technical issues had caused overall severely reduced DNA concentration (see Figure 2). In the two plasma cfDNA (collected at diagnosis and post radiotherapy) from DIPG patient 2, there were no detectable histone mutations.

Next, we screened for the presence of H3 mutations in our cohort of 90 adult patients, utilizing these now-validated highly sensitive ddPCR assays. The clinical parameters of the 89 analyzed adult glioma patients are summarized in Table 3. The additional DHG patient was 26 years of age at diagnosis and his cancer was diagnosed as CNS embryonal tumor (NOS) of right parietal lobe, WHO grade IV.

**Figure 3:**
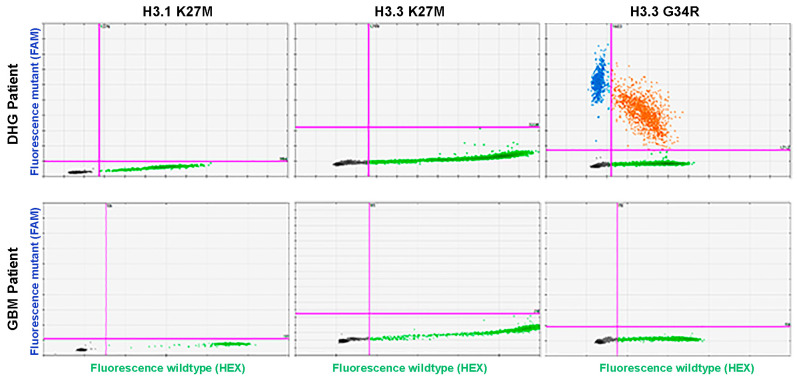
**Histone mutation testing of adult patient tissue DNA**. Depicted are histone mutation assay 2D graphs for the one DHG patient validating H3.3-G34R status and for a patient from the adult glioma cohort with no mutations detected, representative of the overall negative histone mutation status of the 89 glioma patients. Green dots define droplets with wild-type template detected. Blue dots define droplets with the indicated mutant detected. Orange dots define droplets with both mutant and wild-type template detected.

Notably, we confirmed the presence of wild-type DNA in all tissue samples, confirming that the assays performed well. However, our screen did not detect any of the assayed histone mutations within the 89-patient adult glioma cohort while readily confirming H3.3-G34R mutation status for the DHG patient (Figure 3). Given the absence of detectable histone mutations for the adult glioma patients, we opted not to pursue liquid biopsy testing for these mutations in this patient cohort. 

## 4. Discussion

The aim of this study was to establish highly sensitive assays capable of detecting the most prevalent histone mutations traditionally associated with childhood brain cancer and conduct a screen for these mutations in a substantial cohort of age-ranged adult glioma (mainly GBM) patients to establish the prevalence of these relatively rare adult brain cancer mutations in our patient population. 

ddPCR was used to ensure the generation of accurate and reliable assays, as it has been reported to be highly sensitive and reliable for the detection of even low mutant copy numbers in a high wild-type DNA background for various cancer-associated mutations [21,22]. Our assays were developed using synthetic gBlock DNAs and validated using cfDNA equivalents isolated from condition media of cell lines with known histone mutation status. The evaluation of cell-line mutation in “cfDNA” showed 100% specificity with complete mutation detection. Overall, in this study, we assayed 18 patient-derived samples (cell line, CSF, or tissue) with prior established mutation status. The resulting sensitivity/specificity data for these patient samples are as follows: H3.1-H3B-K27M: 100% (CI 15–100%)/100% (CI 79–100%), H3.3-H3F3A-K27M: 100% (CI 54–100%)/100% (CI 75–100%), H3.3-H3F3A-G34R: 100% (CI 15–100%)/100% (CI 79–100%). Additionally, we demonstrated that our assays were able to detect the H3.1-K27M mutation in cfDNA extracted from only 200 µL of CSF from a pediatric patient with DIPG. Other studies tend to use higher volumes (0.5–2 mL) of CSF, if accessible, with one comparable study having an average of 1.74 mL (SD = 1.5) available. The lowest CSF volume in which a mutation was successfully confirmed was 0.7 mL [23,24,25,26,27]. Although we did not detect the mutation in another CSF sample from the same patient, that specific sample was considered of low quality, as we did not detect the wild-type allele either. This indicates that sampling and sample treatment during processing is crucial to maintain high-quality cfDNA. Unsurprisingly, our assay did not detect the mutation in matched plasma samples. As previously reported, and as we confirm here, plasma may be a less promising liquid biopsy for brain cancer due to the blood–brain barrier impeding release of tumor entities into circulation [20]. Instead, our very limited data indicate CSF is a more promising liquid biopsy for this patient group, which is often accessible with an acceptable risk for the patient to obtain important diagnostic information. Notably, this agrees with other reports that CSF is the more reliable liquid biopsy to validate mutations known from brain cancer tumor tissue [28]. Other reports show vastly varying mutation detection efficiency in glioma-patient-plasma-derived cfDNA. One study found *TERT* promoter (*TERTp*) mutations in 4.3% of their cfDNA samples [29], while another reported validation of tissue *TERTp* mutations for 7.3% of patients. Interestingly, that study validated *TERTp* mutations in 100% of patients using CSF cfDNA [30]. A third study, by implementing technical optimizations of plasma volumes, cfDNA extraction, and ddPCR detection managed to validate *TERTp* status in the plasma cfDNA of 68% and 76% of two patient cohorts, respectively [31]. *IDH1* mutation status was validated for 60% of patients using plasma cfDNA [32]. It has been proposed to temporarily disrupt the BBB to improve the cfDNA content of plasma, and a clinical trial assessing this option is currently under way (ClinicalTrials.gov Identifier: NCT05383872). 

Of note, we cannot rule out that plasma quality may have been affected, as we had only 10 mL Streck tubes available for the drawing of 3 mL pediatric blood samples and the ratio of preservative to blood may have been too high, as stipulated in the manufacturer’s documentation. However, since wild-type cfDNA was readily detectable in these samples, we consider it unlikely that cfDNA was affected and, in our experience, we extract high-quality cfDNA with Streck tubes even if circumstances lead to underloading [33]. 

Having proven that our assays reliably detect H3.3-K27M, H3.3-G34R, and H3.1-K27M mutations, we showed that these mutations were not present in the glioma tissue of our adult patient cohort, apart from confirming H3.3-G34R for our DHG patient. This may not be surprising, given that other studies found histone mutations in only small proportions of adult patients. Recently, Bonner et al., in a study of various cancers including 12,743 pediatric and adult tumors, found that 10% of AYA GBM patients, but no older ones, had histone mutations [17]. Thus, if assuming similar numbers, our cohort with only eight AYA patients was too small. However, although age distributions favor histone mutation occurrence in younger patients, there are several reports of older glioma patients with histone mutations [12,34], and H3-K27 mutations in non-midline locations including frontal and temporal lobe [11,13,35,36,37]. The patients in our cohort had a median age of 61 (ranging from 18–85 years) and had tumors primarily localized to the frontal, temporal, and parietal lobes. However, overall, H3 mutations in adult CNS tumors are still relatively understudied, and insufficient data are available to help predict if mutation rates differ for certain ethnic patient populations. Our data give some advance to the hypothesis that histone mutation frequencies may not be related to ethnic background, but larger ethnically diverse cohorts are needed to clarify any ethnic correlation. Regardless, a better understanding of histone mutation frequencies in real-world patient cohorts such as ours remains important, not only to consider patient testing regarding prognostication, but also in case relevant clinical trials emerge in the future. 

Interestingly, a recent international survey study which aimed to identify patterns of care in adult histone mutant gliomas in Australia and the United States reported that only 33% of responders tested all glioma patients for histone mutations routinely, and 26% did not test at all [38]. Further, if these mutations were detected in adult brain cancer, patients were usually diagnosed at a relatively young median age of 32 [39,40], with anatomical tumor localization to the midline (H3-K27) or hemispheric region (H3-G34) [12,40,41]. While this practice has been reported in certain institutions [38], it is crucial to emphasize that solely considering patient age and tumor location, even though they are important factors, falling outside typical ranges may not provide sufficient grounds to exclude the potential presence of histone mutations [38]. The detection of histone mutations may prove important for adult as well as pediatric patients, as histone mutation status appears to co-exist with specific molecular types of cancer, and common co-existence with other biomarkers may open the door for better diagnosis and prognosis, and potentially stratify patients for better targeted therapies and emerging future clinical trials. For instance, H3-K27M mutations are reportedly associated with higher frequencies of *NF1* and *PIK3CA/PIK3R1* mutations, while G34 mutations more commonly co-exist with *CDK4/6* amplification or *CDKN2A/B* loss [34]. The molecular understanding of glioma has recently changed. Mutation screening of *IDH1* is now critical for classification and *TERT* promoter mutations have been strongly implicated as predictive poor outcome biomarkers [10,42,43]. As mentioned, *TERT* promoter mutations can also be successfully screened in blood liquid biopsy [31]. Histone alterations via mutations or modifications, as well as direct DNA modifications, have critical epigenetic consequences and may allow for future development of targeted therapy. Histone modifications have been linked to higher grade glioma [44], while DNA methylation of the *MGMT* gene promoter remains the best predictive biomarker of better response to current adult glioma treatment regimens which include temozolomide [45]. More recently, a seven-gene promoter panel DNA methylation test was proposed to improve the diagnosis of gliomas and may help their molecular characterisation [46]. Given the heterogeneity and better understanding of disease-related molecular pathway hubs [47,48], the emerging evidence points to the value of screening a biomarker signature rather than only alterations of individual molecules to best diagnose glioma. This is also important as certain markers may affect each other. For instance, co-existence with *MGMT* promoter methylation may override the poor prognosis associated with *TERT* promoter mutations [49].

## 5. Conclusions

In summary, we have generated reliable tests for H3.3-K27M, H3.3-G34R, and H3.1-K27M mutations, designed for the testing of tumor tissue and liquid biopsies. We confirmed that these mutations are detectable in cfDNA equivalents from cultured cells as well as from patient CSF, while our limited data confirm poor ctDNA content within cfDNA isolated from brain cancer patient plasma. We further confirmed the rarity of histone mutations in adult brain cancer patients, with none found in our Southwestern Sydney cohort of 89 glioma patients. Our data add to the current body of literature and have implications for developing biomarker testing for glioma patients. The future likely lies in the development of prognostic and predictive panels of glioma biomarkers, and due to the lower costs associated with better technologies, testing for rare histone mutations may remain a relevant component of such testing, even in adult patients, to provide the best possible care to each patient.

## Figures and Tables

**Table 1:** Primers, probes, and gBlocks.

Primers and Probes			
	H3.1-K27M	H3.3-K27M	H3.3-G34R
Forward primer	5′-AACAGACAGCTCGGAAATC-3′	5′-AAATCGACCGGTGGTAAAGC-3′	
Reverse primer	5′-TAACGGTGAGGCTTTTTCA-3′	5′-AATACCTGTAACGATGAGGT-3′	
Wild-type probe	5′-HEX-CTCGCAAGAGCGCGCCG-BQ1-3′	5′-HEX-CACTCTTGCGAGCGGCTT-BQ1-3′	5′-HEX-TCTACTGGAGGGGTGAAGAA-BQ1-3′
Mutant probe	5′-FAM-CTCGCA**T**GAGCGCGCCG-BQ1-3′	5′-FAM-CACTC**A**TGCGAGCGGCTT-BQ1-3′	5′-FAM-TCTACTGGA**A**GGGTGAAGAA-BQ1-3′
	**gBlock Sequence (NIH Nucleotide GenBank ID)**		
H3.1-K27M	AF531275.1 Range 391–537; base 493 changed (A to T)		
H3.3-K27M	NG065173.1 Range 6638–6809; base 6729 changed (A to T)		
H3.3-G34R	NG065173.1 Range 6638–6809; base 6749 changed (G to A)		

Note: base alterations are highlighted in **bold with underscore**. Abbreviations: HEX—Hexachloro-Fluorescein fluorescent dye; BQ1—Black Hole Quencher-1; FAM—Carboxyfluorescein fluorescent dye; gBlock—Synthetic double-stranded DNA fragments.

**Table 2:** Validation of mutation detection from cell line conditioned media.

Cell Line	Mutation Detected *
SU-DIPG21	H3.1 H3B K27M
HSJD-GBM2	H3.3 H3F3A G34R
SU-DIPG24	H3.3 H3F3A K27M
HSJD-DIPG007	H3.3 H3F3A K27M
P00208	H3.3 H3F3A K27M
RA055	H3.3 H3F3A K27M
SU-DIPG17	H3.3 H3F3A K27M
SU-DIPG6	H3.3 H3F3A K27M
RA021	wild-type
VUMC-DIPG10	wild-type
P000106	wild-type
P001003	wild-type
P001105	wild-type
P001302	wild-type
P001802	wild-type
RA034	wild-type

* All detected mutations validated known cell line mutation data; wild-type was found regarding all three tested variants.

**Table 3:** Clinical characteristics of 89 glioma patients.

Characteristics	Patients, *n* (%)							
**Age group**	18–39 years		40–65 years		66–85 years		Total	
**No. of patients**	8		41		40		89	
**Mean age in years at diagnosis [Range]**	30 [18–36]		55 [43–65]		73 [66–85]		61 [18–85]	
**Sex**								
Male	8	(100)	26	(63.4)	25	(63.5)	59	(66.3)
Female	0	(0)	15	(36.6)	15	(37.5)	30	(33.7)
**Grade**								
WHO grade 4	7	(87.5)	40	(97.6)	40	(100)	87	(97.8)
WHO grade 2 or 3	1	(12.5)	1	(2.4)	0	(0)	2	(2.2)
**Surgical Resection**								
GTR	3	(37.5)	16	(39)	12	(30)	31	(34.8)
STR	5	(62.5)	23	(56.1)	22	(55)	50	(56.2)
Biopsy	0	(0)	2	(4.9)	6	(15)	8	(9)
**Radiation**								
Yes	8	(100)	35	(85.4)	24	(60)	67	(75.3)
No		(0)	6	(14.6)	16	(40)	22	(24.7)
**Concurrent TMZ**								
Yes	8	(100)	36	(87.8)	21	(52.5)	65	(73)
No		(0)	5	(12.2)	19	(47.5)	24	(27)
**Tumour Location**								
Frontal	4	(50)	19	(46.3)	11	(27.5)	34	(38.2)
Parietal	2	(25)	5	(12.2)	6	(15)	13	(14.6)
Temporal	1	(12.5)	10	(24.4)	15	(37.5)	26	(29.2)
Other	1	(12.5)	7	(17.1)	8	(20)	16	(18)
**Mean overall survival in days [Range]**	1067 [429–2100]		602 [23–2879]		300 [27–1430]		493 [23–2879]	

GTR—gross total resection; STR—subtotal resection; TMZ—temozolomide.

## Data Availability

All data are available in the manuscript, ddPCR raw data are made available upon reasonable request.

## References

[1] Crowell C., Mata-Mbemba D., Bennett J., Matheson K., Mackley M., Perreault S., Erker C. (2022). Systematic review of diffuse hemispheric glioma, H3 G34-mutant: Outcomes and associated clinical factors. Neurooncol. Adv..

[2] Vuong H.G., Ngo T.N.M., Le H.T., Dunn I.F. (2022). The prognostic significance of HIST1H3B/C and H3F3A K27M mutations in diffuse midline gliomas is influenced by patient age. J. Neurooncol..

[3] Adhikari S., Bhutada A.S., Ladner L., Cuoco J.A., Entwistle J.J., Marvin E.A., Rogers C.M. (2023). Prognostic Indicators for H3K27M-Mutant Diffuse Midline Glioma: A Population-Based Retrospective Surveillance, Epidemiology, and End Results Database Analysis. World Neurosurg..

[4] Schwartzentruber J., Korshunov A., Liu X.Y., Jones D.T., Pfaff E., Jacob K., Sturm D., Fontebasso A.M., Quang D.A., Tonjes M. (2012). Driver mutations in histone H3.3 and chromatin remodelling genes in paediatric glioblastoma. Nature.

[5] Wu G., Broniscer A., McEachron T.A., Lu C., Paugh B.S., Becksfort J., Qu C., Ding L., Huether R., Parker M. (2012). Somatic histone H3 alterations in pediatric diffuse intrinsic pontine gliomas and non-brainstem glioblastomas. Nat. Genet..

[6] Behjati S., Tarpey P.S., Presneau N., Scheipl S., Pillay N., Van Loo P., Wedge D.C., Cooke S.L., Gundem G., Davies H. (2013). Distinct H3F3A and H3F3B driver mutations define chondroblastoma and giant cell tumor of bone. Nat. Genet..

[7] Lehnertz B., Zhang Y.W., Boivin I., Mayotte N., Tomellini E., Chagraoui J., Lavallee V.P., Hebert J., Sauvageau G. (2017). H3(K27M/I) mutations promote context-dependent transformation in acute myeloid leukemia with RUNX1 alterations. Blood.

[8] Papillon-Cavanagh S., Lu C., Gayden T., Mikael L.G., Bechet D., Karamboulas C., Ailles L., Karamchandani J., Marchione D.M., Garcia B.A. (2017). Impaired H3K36 methylation defines a subset of head and neck squamous cell carcinomas. Nat. Genet..

[9] Lowe B.R., Maxham L.A., Hamey J.J., Wilkins M.R., Partridge J.F. (2019). Histone H3 Mutations: An Updated View of Their Role in Chromatin Deregulation and Cancer. Cancers.

[10] Louis D.N., Perry A., Wesseling P., Brat D.J., Cree I.A., Figarella-Branger D., Hawkins C., Ng H.K., Pfister S.M., Reifenberger G. (2021). The 2021 WHO Classification of Tumors of the Central Nervous System: A summary. Neuro Oncol..

[11] Chia N., Wong A., Teo K., Tan A.P., Vellayappan B.A., Yeo T.T., Oh S.Y., Tan C.L. (2021). H3K27M-mutant, hemispheric diffuse glioma in an adult patient with prolonged survival. Neurooncol. Adv..

[12] Meyronet D., Esteban-Mader M., Bonnet C., Joly M.O., Uro-Coste E., Amiel-Benouaich A., Forest F., Rousselot-Denis C., Burel-Vandenbos F., Bourg V. (2017). Characteristics of H3 K27M-mutant gliomas in adults. Neuro Oncol..

[13] Nakata S., Nobusawa S., Yamazaki T., Osawa T., Horiguchi K., Hashiba Y., Yaoita H., Matsumura N., Ikota H., Hirato J. (2017). Histone H3 K27M mutations in adult cerebellar high-grade gliomas. Brain Tumor Pathol..

[14] AlRayahi J., Alwalid O., Mubarak W., Maaz A.U.R., Mifsud W. (2023). Pediatric Brain Tumors in the Molecular Era: Updates for the Radiologist. Semin. Roentgenol..

[15] Di Nunno V., Franceschi E., Gatto L., Tosoni A., Bartolini S., Brandes A.A. (2023). How to treat histone 3 altered gliomas: Molecular landscape and therapeutic developments. Expert. Rev. Clin. Pharmacol..

[16] Solomon D.A., Wood M.D., Tihan T., Bollen A.W., Gupta N., Phillips J.J., Perry A. (2016). Diffuse Midline Gliomas with Histone H3-K27M Mutation: A Series of 47 Cases Assessing the Spectrum of Morphologic Variation and Associated Genetic Alterations. Brain Pathol..

[17] Bonner E.R., Dawood A., Gordish-Dressman H., Eze A., Bhattacharya S., Yadavilli S., Mueller S., Waszak S.M., Nazarian J. (2023). Pan-cancer atlas of somatic core and linker histone mutations. NPJ Genom. Med..

[18] Burkart M., Sanford S., Dinner S., Sharp L., Kinahan K. (2019). Future health of AYA survivors. Pediatr. Blood Cancer.

[19] Lasocki A., Abdalla G., Chow G., Thust S.C. (2022). Imaging features associated with H3 K27-altered and H3 G34-mutant gliomas: A narrative systematic review. Cancer Imaging.

[20] Sareen H., Garrett C., Lynch D., Powter B., Brungs D., Cooper A., Po J., Koh E.S., Vessey J.Y., McKechnie S. (2020). The Role of Liquid Biopsies in Detecting Molecular Tumor Biomarkers in Brain Cancer Patients. Cancers.

[21] Wolter M., Felsberg J., Malzkorn B., Kaulich K., Reifenberger G. (2022). Droplet digital PCR-based analyses for robust, rapid, and sensitive molecular diagnostics of gliomas. Acta Neuropathol. Commun..

[22] Ding P.N., Becker T., Bray V., Chua W., Ma Y., Xu B., Lynch D., de Souza P., Roberts T. (2019). Plasma next generation sequencing and droplet digital PCR-based detection of epidermal growth factor receptor (EGFR) mutations in patients with advanced lung cancer treated with subsequent-line osimertinib. Thorac. Cancer.

[23] Martinez-Ricarte F., Mayor R., Martinez-Saez E., Rubio-Perez C., Pineda E., Cordero E., Cicuendez M., Poca M.A., Lopez-Bigas N., Ramon Y.C.S. (2018). Molecular Diagnosis of Diffuse Gliomas through Sequencing of Cell-Free Circulating Tumor DNA from Cerebrospinal Fluid. Clin. Cancer Res..

[24] Li D., Bonner E.R., Wierzbicki K., Panditharatna E., Huang T., Lulla R., Mueller S., Koschmann C., Nazarian J., Saratsis A.M. (2021). Standardization of the liquid biopsy for pediatric diffuse midline glioma using ddPCR. Sci. Rep..

[25] Panditharatna E., Kilburn L.B., Aboian M.S., Kambhampati M., Gordish-Dressman H., Magge S.N., Gupta N., Myseros J.S., Hwang E.I., Kline C. (2018). Clinically Relevant and Minimally Invasive Tumor Surveillance of Pediatric Diffuse Midline Gliomas Using Patient-Derived Liquid Biopsy. Clin. Cancer Res..

[26] Garcia-Romero N., Carrion-Navarro J., Areal-Hidalgo P., Ortiz de Mendivil A., Asensi-Puig A., Madurga R., Nunez-Torres R., Gonzalez-Neira A., Belda-Iniesta C., Gonzalez-Rumayor V. (2019). BRAF V600E Detection in Liquid Biopsies from Pediatric Central Nervous System Tumors. Cancers.

[27] Izquierdo E., Proszek P., Pericoli G., Temelso S., Clarke M., Carvalho D.M., Mackay A., Marshall L.V., Carceller F., Hargrave D. (2021). Droplet digital PCR-based detection of circulating tumor DNA from pediatric high grade and diffuse midline glioma patients. Neurooncol. Adv..

[28] Friedman J.S., Hertz C.A.J., Karajannis M.A., Miller A.M. (2022). Tapping into the genome: The role of CSF ctDNA liquid biopsy in glioma. Neurooncol. Adv..

[29] Fontanilles M., Marguet F., Beaussire L., Magne N., Pepin L.F., Alexandru C., Tennevet I., Hanzen C., Langlois O., Jardin F. (2020). Cell-free DNA and circulating TERT promoter mutation for disease monitoring in newly-diagnosed glioblastoma. Acta Neuropathol. Commun..

[30] Juratli T.A., Stasik S., Zolal A., Schuster C., Richter S., Daubner D., Juratli M.A., Thowe R., Hennig S., Makina M. (2018). TERT Promoter Mutation Detection in Cell-Free Tumor-Derived DNA in Patients with IDH Wild-Type Glioblastomas: A Pilot Prospective Study. Clin. Cancer Res..

[31] Muralidharan K., Yekula A., Small J.L., Rosh Z.S., Kang K.M., Wang L., Lau S., Zhang H., Lee H., Bettegowda C. (2021). TERT Promoter Mutation Analysis for Blood-Based Diagnosis and Monitoring of Gliomas. Clin. Cancer Res..

[32] Boisselier B., Gallego Perez-Larraya J., Rossetto M., Labussiere M., Ciccarino P., Marie Y., Delattre J.Y., Sanson M. (2012). Detection of IDH1 mutation in the plasma of patients with glioma. Neurology.

[33] Kahana-Edwin S., Cain L.E., Karpelowsky J. (2021). Roadmap to Liquid Biopsy Biobanking from Pediatric Cancers-Challenges and Opportunities. Biopreserv. Biobank..

[34] Williams E.A., Brastianos P.K., Wakimoto H., Zolal A., Filbin M.G., Cahill D.P., Santagata S., Juratli T.A. (2023). A comprehensive genomic study of 390 H3F3A-mutant pediatric and adult diffuse high-grade gliomas, CNS WHO grade 4. Acta Neuropathol..

[35] Wang L., Li Z., Zhang M., Piao Y., Chen L., Liang H., Wei Y., Hu Z., Zhao L., Teng L. (2018). H3 K27M-mutant diffuse midline gliomas in different anatomical locations. Hum. Pathol..

[36] Lopez G., Oberheim Bush N.A., Berger M.S., Perry A., Solomon D.A. (2017). Diffuse non-midline glioma with H3F3A K27M mutation: A prognostic and treatment dilemma. Acta Neuropathol. Commun..

[37] Onishi S., Ohba S., Kuraoka K., Kurashige T., Sugiyama K., Yamasaki F. (2022). Molecular and clinical characterization of H3 K27M-mutant "non-midline" glioblastoma: A case report and literature review. Neurocirugia.

[38] Yuile A., Khasraw M., Low J.T., Walsh K.M., Lipp E., Sy J., Satgunaseelan L., Kastelan M.A., De Silva M., Lee A. (2022). Patterns of care in adult histone mutant gliomas: Results of an international survey. Neurooncol. Pract..

[39] Zhao Y., Chen Y., Wang L., Gao Y., Xu J. (2023). The clinicopathological features and prognosis of multifocal high-grade gliomas in adults with H3F3A mutation. Neurosciences.

[40] Schulte J.D., Buerki R.A., Lapointe S., Molinaro A.M., Zhang Y., Villanueva-Meyer J.E., Perry A., Phillips J.J., Tihan T., Bollen A.W. (2020). Clinical, radiologic, and genetic characteristics of histone H3 K27M-mutant diffuse midline gliomas in adults. Neurooncol. Adv..

[41] Chen C.C.L., Deshmukh S., Jessa S., Hadjadj D., Lisi V., Andrade A.F., Faury D., Jawhar W., Dali R., Suzuki H. (2020). Histone H3.3G34-Mutant Interneuron Progenitors Co-opt PDGFRA for Gliomagenesis. Cell.

[42] Sledzinska P., Bebyn M.G., Furtak J., Kowalewski J., Lewandowska M.A. (2021). Prognostic and Predictive Biomarkers in Gliomas. Int. J. Mol. Sci..

[43] Powter B., Jeffreys S.A., Sareen H., Cooper A., Brungs D., Po J., Roberts T., Koh E.S., Scott K.F., Sajinovic M. (2021). Human TERT promoter mutations as a prognostic biomarker in glioma. J. Cancer Res. Clin. Oncol..

[44] Ozair A., Bhat V., Alisch R.S., Khosla A.A., Kotecha R.R., Odia Y., McDermott M.W., Ahluwalia M.S. (2023). DNA Methylation and Histone Modification in Low-Grade Gliomas: Current Understanding and Potential Clinical Targets. Cancers.

[45] Hegi M.E., Genbrugge E., Gorlia T., Stupp R., Gilbert M.R., Chinot O.L., Nabors L.B., Jones G., Van Criekinge W., Straub J. (2019). MGMT Promoter Methylation Cutoff with Safety Margin for Selecting Glioblastoma Patients into Trials Omitting Temozolomide: A Pooled Analysis of Four Clinical Trials. Clin. Cancer Res..

[46] Majchrzak-Celinska A., Dybska E., Barciszewska A.M. (2020). DNA methylation analysis with methylation-sensitive high-resolution melting (MS-HRM) reveals gene panel for glioma characteristics. CNS Neurosci. Ther..

[47] Weiser A., Sanchez Bergman A., Machaalani C., Bennett J., Roth P., Reimann R.R., Nazarian J., Guerreiro Stucklin A.S. (2023). Bridging the age gap: A review of molecularly informed treatments for glioma in adolescents and young adults. Front. Oncol..

[48] Yin X., Wu Q., Hao Z., Chen L. (2022). Identification of novel prognostic targets in glioblastoma using bioinformatics analysis. Biomed. Eng. Online.

[49] Vuong H.G., Nguyen T.Q., Ngo T.N.M., Nguyen H.C., Fung K.M., Dunn I.F. (2020). The interaction between TERT promoter mutation and MGMT promoter methylation on overall survival of glioma patients: A meta-analysis. BMC Cancer.

